# Beziehungsqualität von Hochaltrigen in Deutschland – Untersuchung der Assoziation mit depressiven Symptomen und Einsamkeit

**DOI:** 10.1007/s00103-026-04277-9

**Published:** 2026-07-29

**Authors:** André Hajek, Hans-Helmut König

**Affiliations:** Institut für Gesundheitsökonomie und Versorgungsforschung, Hamburg Center for Health Economics, Universitätsklinikum Hamburg-Eppendorf, Martinistraße 52, 20246 Hamburg, Deutschland

**Keywords:** Depression, Hochaltrig, Familie, Freunde, Beziehungsqualität, Depression, Oldest old, Family, Friends, Relationship quality

## Abstract

**Einleitung:**

Die Assoziation von Beziehungsqualität zu Familie und Freunden mit depressiven Symptomen und Einsamkeit wurde bei Hochaltrigen bislang kaum untersucht. Ziel der Studie war es, diese Forschungslücke zu adressieren.

**Methoden:**

Datengrundlage war Welle 8 des Deutschen Alterssurveys (Dezember 2022 bis Juni 2023), einer bundesweit repräsentativen Stichprobe von zu Hause lebenden Personen in der zweiten Lebenshälfte. In die Analysen wurden hochaltrige Personen ab 80 Jahren einbezogen (analytische Stichprobe: *n* = 810). Einsamkeit wurde mit der De-Jong-Gierveld-Skala erfasst, depressive Symptome mit der Kurzform der Allgemeinen Depressionsskala (ADS-K). Die Beziehungsqualität zu Familie und Freunden wurde jeweils mittels Single-Items erhoben. Die Auswertung erfolgte anhand multipler linearer Regressionsmodelle.

**Ergebnisse:**

Eine hohe Beziehungsqualität zur Familie (im Vergleich zu einer geringen Beziehungsqualität) war weder mit depressiven Symptomen noch mit Einsamkeit statistisch signifikant assoziiert. Im Gegensatz dazu war eine hohe Beziehungsqualität zu Freunden (im Vergleich zu einer niedrigen Beziehungsqualität) statistisch signifikant mit weniger depressiven Symptomen (β = −3,71, *p* < 0,01) und geringerer Einsamkeit (β = −0,28, *p* < 0,01) assoziiert.

**Diskussion:**

Unsere Ergebnisse zeigen eine Assoziation einer hohen Beziehungsqualität zu Freunden mit weniger depressiven Symptomen und weniger Einsamkeit bei Hochaltrigen in Deutschland. Künftige Studien sollten die zugrunde liegenden Mechanismen für die identifizierten Assoziationen näher untersuchen.

**Zusatzmaterial online:**

Zusätzliche Informationen sind in der Online-Version dieses Artikels (https://doi.org/10.1007/s00103-026-04277-9) enthalten.

## Abstract (en)

**Background:**

To date, there is restricted knowledge with regard to the association of relationship quality with family and friends and depressive symptoms and loneliness among the oldest old. Thus, we aimed to address this knowledge gap.

**Methods:**

Data were taken from wave 8 of the German Ageing Survey (December 2022 to June 2023), a nationally representative sample of community-dwelling individuals in their second half of life. We restricted our analytical sample (*n* = 810 respondents) to individuals aged 80 years and over (i.e., oldest old). Loneliness was measured using the De Jong Gierveld tool. The Center for Epidemiologic Studies Depression Scale (CES-D) was used to measure depressive symptoms. Multiple linear regression models were estimated. Both the quality of relationships with family and friends was measured via single-item tools. Multiple linear regression models were used.

**Results:**

A high quality of relationship with family (compared to a low-quality relationship) was neither statistically significantly associated with depressive symptoms nor with loneliness. Contrary, a high quality of relationship with friends (compared to a low-quality relationship) was significantly associated with fewer depressive symptoms (β = −3.71, *p* < 0.01) and lower loneliness levels (β = −0.28, *p* < 0.01).

**Conclusions:**

Our findings revealed an association of a high quality of relationship with friends and fewer depressive symptoms and lower loneliness scores among the oldest old in Germany. Upcoming research should elucidate the underlying mechanisms of the identified associations.

## Einleitung

Es ist davon auszugehen, dass die Zahl hochaltriger Menschen (d. h. 80 Jahre und älter) in Deutschland in den nächsten Jahrzehnten im Zuge der demografischen Alterung immer stärker zunehmen wird [1]. In diesem Alter können viele kritische Lebensereignisse auftreten, wie bspw. die deutliche Verschlechterung der Gesundheit oder auch der Tod von Familienangehörigen und Freunden [2,3]. Derartige Faktoren können dazu führen, dass depressive Symptome (z. B. Energieverlust, Hoffnungslosigkeit, Interessenverlust, Freudlosigkeit oder auch Schlafstörungen) und Einsamkeit (wahrgenommener Unterschied zwischen den realen und den gewünschten sozialen Beziehungen [4]) zunehmen [5]. Dies ist von großer Bedeutung, da depressive Symptome und Einsamkeit ihrerseits mit erhöhter Sterblichkeit, chronischen Erkrankungen sowie dem Wunsch bzw. der Erwartung eines früheren Todes assoziiert sind [6–9]. Deshalb ist es wesentlich, die Determinanten von depressiven Symptomen und Einsamkeit bei Hochaltrigen besser zu verstehen.

Depressive Symptome sind mit Einsamkeit positiv korreliert, können aber auch unabhängig voneinander auftreten. Viele Studien haben bspw. schon die Determinanten von depressiven Symptomen und Einsamkeit im Alter untersucht. Faktoren wie die Wohnsituation, der Familienstand, sensorische Beeinträchtigungen oder die subjektive Gesundheit können wesentlich für diese Zielgrößen im Alter sein (als Übersicht: [10–12]). Allerdings können sich die Determinanten auch unterscheiden. Eine frühere Studie bei Hochaltrigen in Deutschland hat bspw. gezeigt, dass Vermögensarmut nur mit mehr depressiven Symptomen assoziiert ist, wohingegen ein größeres Netzwerk lediglich mit weniger Einsamkeit assoziiert ist [13].

Frühere Studien konnten auch Zusammenhänge insbesondere der Beziehungsqualität zu Freunden und den genannten Outcomes aufzeigen. Die bisherigen Studien fokussierten sich allerdings meist eher auf Kinder und Heranwachsende [14–16]. Derartige Studien bei älteren Populationen existieren kaum [17,18], zeigen aber vorläufige Hinweise für entsprechende Zusammenhänge auch im Alter auf.

Zusammenfassend gibt es bisher kaum Erkenntnisse zur Assoziation von Beziehungsqualität zu Familie und Freunden mit depressiven Symptomen und Einsamkeit bei Hochaltrigen global betrachtet. Explizit fehlen dazu Studien zu Hochaltrigen in Deutschland. Deshalb zielt diese Arbeit darauf ab, diese Forschungslücke zu adressieren. Die Erkenntnisse dieser Studie sind wichtig, um Einsamkeit und depressive Symptome bei Hochaltrigen in Deutschland besser verstehen zu können. Wir vermuten, dass sowohl die Beziehungsqualität zu Familie als auch zu Freunden mit weniger depressiven Symptomen und geringeren Einsamkeitsleveln assoziiert ist, da beide Bereiche vermutlich lebenslange Bindungen reflektieren. Sie sind wahrscheinlich geprägt von großer Vertrautheit. Eine gute Beziehung zu Familie und Freunden könnte zur sozialen Integration und emotionalen Unterstützung beitragen [19]. Vorab hatten wir keine konkreten Vermutungen dahin gehend, ob die Beziehungsqualität zur Familie stärker mit den Outcomes assoziiert ist als die zu Freunden.

## Methoden

### Stichprobe.

Für diese Studie wurden Daten der neuesten Welle 8 des Deutschen Alterssurveys (DEAS) genutzt. Generell fokussiert sich die DEAS-Studie bevölkerungsrepräsentativ auf zu Hause lebende Individuen in der zweiten Lebenshälfte (d. h. ab 40 Jahren). Die DEAS-Studie wird aus Mitteln des Bundesministeriums für Bildung, Familie, Senioren, Frauen und Jugend (BMBFSFJ) gefördert.

Ein besseres Verständnis der zweiten Lebenshälfte ist ein zentrales Ziel der DEAS-Studie. Die Datenerhebung der 8. Welle erfolgte von Ende 2022 bis Mitte 2023. Als Befragungsmethoden wurden CAPI-Interviews (Computer Assisted Personal Interviewing) und CATI-Interviews (Computer Assisted Telephone Interviewing) eingesetzt. Zusätzlich wurde im Anschluss eine schriftliche Befragung durchgeführt, bei der eher sensible Themen – wie Einsamkeit – erfasst wurden. Sprache für Interviews und Fragebögen war jeweils ausschließlich Deutsch.

Die Rücklaufquote in Welle 8 betrug ca. 62 %. In Welle 8 wurden lediglich Individuen befragt, die schon in mindestens einer früheren Welle befragt wurden. Da unser Erkenntnisinteresse sich in dieser Studie auf Hochaltrige fokussierte, haben wir unsere Stichprobe auf Personen ab 80 Jahren beschränkt. Die analytische Stichprobe umfasste 810 Individuen (mit depressiven Symptomen als Outcome; 797 Individuen mit Einsamkeit als Outcome). Diese Stichprobe bezieht sich also auf Individuen, die in dem adjustierten Regressionsmodell inkludiert sind. Weitere Details zur DEAS-Studie finden sich bei [20].

### Outcomes.

*Depressive Symptome* fungierten als erste abhängige Variable. Diese wurden mithilfe der 15-Item-Version der Allgemeinen Depressions-Skala (ADS‑K; Englisch: Center for Epidemiologic Studies Depression Scale, CES‑D [21]) gemessen. Diese erfasst unterschiedliche Symptome (z. B. affektive Symptome, kognitive Einschränkungen, Schlaf oder körperliche Symptome). Ein exemplarisches Item ist: „Während der letzten Woche war ich deprimiert/niedergeschlagen“ (1 = Selten oder überhaupt nicht, also weniger als 1 Tag; 2 = Manchmal, also an 1 bis 2 Tagen; 3 = Öfters, also an 3 bis 4 Tagen; 4 = Meistens oder die ganze Zeit, also an 5 bis 7 Tagen). Ein Summenwert wurde aus den 15 Items gebildet (2 Items wurden davor rekodiert). Die Skala variiert dabei von 0 bis 45. Höhere Werte entsprechen dabei mehr depressiven Symptomen. Cronbachs Alpha war 0,86 (McDonalds Omega war 0,87). Die Skala wird häufig eingesetzt [22] und hat sehr gute psychometrische Eigenschaften [23].

*Einsamkeit* war die zweite abhängige Variable. Diese wurde mittels der De-Jong-Gierveld-(DJG-)Einsamkeitsskala (6-Item-Version; [24]) gemessen. Ein exemplarisches Item ist: „Ich vermisse Leute, bei denen ich mich wohlfühle“ (1 = trifft gar nicht zu; 2 = trifft eher nicht zu; 3 = trifft eher zu; 4 = trifft genau zu). Vor Bildung des Mittelwertes wurden 3 Items rekodiert. Die Skala variiert zwischen 1 und 4. Höhere Werte spiegeln eine höhere Einsamkeit wider. Cronbachs Alpha betrug 0,84 (McDonalds Omega war ebenfalls 0,84). Auch diese Skala wird vielfach verwendet [11] und weist gute psychometrische Charakteristika auf [24].

### Unabhängige Variablen.

Die beiden Variablen *Qualität der Beziehung zur Familie* und *Qualität der Beziehung zu Freunden* dienten als die wesentlichen unabhängigen Variablen. Diese wurden direkt mithilfe von Single-Items abgefragt: „Einmal insgesamt betrachtet, wie bewerten Sie Ihre derzeitige Beziehung zu Ihrer Familie?“ sowie: „Wie bewerten Sie Ihr derzeitiges Verhältnis zu Ihren Freunden und Bekannten?“ Antwortoptionen waren jeweils: 1 = Sehr gut; 2 = Gut; 3 = Mittel; 4 = Schlecht; 5 = Sehr schlecht (und „Trifft nicht zu“ als Ausweichoption). Aufgrund der Verteilung haben wir beide Variablen dichotomisiert (0 = sehr schlecht, schlecht oder mittel, 1 = gut oder sehr gut). Die Ausweichoption „trifft nicht zu“ wurde exkludiert.

### Kovariaten.

Basierend auf eigenen Überlegungen und früheren Studien [10–12] wurden die Kovariaten für das Regressionsmodell gewählt. Beispielsweise kann die familiäre Situation sowohl die Beziehungsqualität zu Familien/Freunden als auch die Outcomes bei Hochaltrigen in Deutschland tangieren [25,26]. Vergleichbare Beziehungen könnten auch für lebensstil- bzw. gesundheitsbezogene Faktoren vorliegen [25,26].

Die folgenden sozioökonomischen Größen wurden genutzt: Alter (in Jahren), Geschlecht (Mann; Frau), Familienstand (5 Kategorien: verheiratet, zusammenlebend; verheiratet, getrennt lebend; geschieden; verwitwet; ledig), Bildungsstand (basierend auf der ISCED (International Standard Classification of Education)-Klassifikation [27] mit 3 Kategorien: niedrig, mittel, hoch) und das Haushaltsnettoäquivalenzeinkommen in Euro. Folgende Lebensstilfaktoren wurden im Regressionsmodell berücksichtigt: Rauchverhalten (4 Kategorien: ja, täglich; ja, gelegentlich; nein, nicht mehr; nein, noch nie), Häufigkeit alkoholischer Getränke und Häufigkeit sportlicher Aktivitäten (in beiden Fällen mit 6 Kategorien: täglich; mehrmals die Woche; einmal die Woche; 1–3 × im Monat; seltener; nie). Bezüglich der gesundheitsbezogenen Faktoren wurden folgende Faktoren in das Regressionsmodell aufgenommen: subjektive Gesundheit mithilfe eines Single-Item-Tools (mit 5 Stufen: 1 = sehr gut; 2 = gut; 3 = mittel; 4 = schlecht; 5 = sehr schlecht) sowie die Anzahl an chronischen Krankheiten (als Count-Score von 0 bis 11 chronische Krankheiten, z. B. Herz- oder Kreislauferkrankung, Krebs, Blasenleiden oder Diabetes). Vertiefende Informationen finden sich hier [28].

### Statistische Analyse.

Die analytische Stichprobe wurde zunächst beschrieben (Mittelwerte und Standardabweichung (SD) bzw. absolute und relative Häufigkeit). In der Folge wurde die Assoziation von Beziehungsqualität zu Familie und Freunden mit depressiven Symptomen und Einsamkeit bei Hochaltrigen mithilfe von unadjustierten und adjustierten linearen Regressionen geschätzt. Es wurde zunächst ein fallweiser Ausschluss gewählt, um fehlende Werte zu adressieren. In der Hauptanalyse wurde ein FIML-Ansatz (Full Information Maximum Likelihood; [29]) genutzt, um fehlende Werte zu adressieren. Solche zusätzlichen Analysen werden dargestellt, um die Robustheit der Ergebnisse darzulegen. Cluster-robuste Standardfehler (die auf der Ebene der Primäreinheiten der Stichprobe, d. h. die gezogenen Gemeinden, clustern) wurden berechnet. Gewichte wurden genutzt [30], um das komplexe Stichprobendesign der DEAS-Studie [20] zu berücksichtigen. Bezüglich der Effektgrößen (in den linearen Regressionen) wurde auch das partielle Eta^2^ berechnet, um die praktische Relevanz einordnen zu können (gängige Klassifikation: 0,01: kleine Effektgröße, 0,06: mittlere Effektgröße, 0,14: große Effektgröße [31]).

McDonald’s Omega wurde mithilfe von „omegacoef“ [32] berechnet. Für die statistische Signifikanz wurde α = 0,05 gewählt. StataNow 19,5 MP—Parallel Edition (Stata Corp., College Station, Texas) wurde für die Datenanalyse verwendet.

## Ergebnisse

### Stichprobenbeschreibung.

Die Beschreibung der analytischen Stichprobe (gewichtet) findet sich in Tab. 1. Im Mittel betrug das Alter 83,7 Jahre (SD: 2,7 Jahre; von 80 bis 90 Jahre). Insgesamt waren 65,5 % der Befragten weiblich. Der Mittelwert auf der Einsamkeitsskala lag bei 1,8 (SD: 0,5), der Mittelwert auf der Skala zur Erfassung depressiver Symptome war 8,0 (SD: 6,6). Die Beziehungsqualität war größtenteils hoch (Beziehungsqualität bzgl. der Familie: 87,4 % hoch; Beziehungsqualität bzgl. der Freunde: 89,6 % hoch). Zusätzliche Details sind in Tab. 1 dargestellt.

**Tab. 1:** Beschreibung der analytischen Stichprobe (*n* = 810, gewichtet)

Variablen	Ausprägung	Mittelwert (SD)/*n* (%)
Alter	In Jahren	83,7 (2,7)
Geschlecht	Mann	280 (34,5 %)
	Frau	530 (65,5 %)
Familienstand	Verheiratet, zusammenlebend	367 (45,3 %)
	Verheiratet, getrenntlebend	3 (0,4 %)
	Geschieden	61 (7,6 %)
	Verwitwet	355 (43,9 %)
	Ledig	23 (2,9 %)
Bildung (ISCED-Klassifikation)	Niedrig (ISCED 0 bis 2)	293 (36,2 %)
	Mittel (ISCED 3 bis 4)	390 (48,2 %)
	Hoch (ISCED 5 bis 6)	126 (15,6 %)
Monatliches Haushaltsnettoäquivalenzeinkommen	In Euro	1993,6 (934,1)
Rauchverhalten	Ja, täglich	39 (4,9 %)
	Ja, gelegentlich	17 (2,1 %)
	Nein, nicht mehr	316 (39,4 %)
	Nein, habe noch nie geraucht	431 (53,7 %)
Häufigkeit: Sport	Täglich	82 (10,1 %)
	Mehrmals pro Woche	106 (13,1 %)
	Einmal pro Woche	70 (8,7 %)
	1–3 × im Monat	27 (3,3 %)
	Seltener	111 (13,7 %)
	Nie	414 (51,1 %)
Häufigkeit: alkoholische Getränke	Täglich	72 (9,0 %)
	Mehrmals pro Woche	135 (16,8 %)
	Einmal pro Woche	112 (13,9 %)
	1–3 × im Monat	45 (5,7 %)
	Seltener	240 (29,9 %)
	Nie	199 (24,8 %)
Subjektiver Gesundheitszustand	(von 1 = sehr gut bis 5 = sehr schlecht)	2,7 (0,8)
Anzahl chronische Erkrankungen	(von 0 bis 11 chronische Erkrankungen)	3,9 (2,2)
Beziehungsqualität^a^: Familie	Niedrig	101 (12,6 %)
	Hoch	701 (87,4 %)
Beziehungsqualität^a^: Freunde	Niedrig	82 (10,4 %)
	Hoch	702 (89,6 %)
Depressive Symptome	(ADS‑K; 0 bis 45: je höher der Wert, desto mehr depressive Symptome)	8,0 (6,6)
Einsamkeit	(DJG-Skala; 1 bis 4: je höher der Wert, desto höher das Einsamkeitslevel)	1,8 (0,5)

^a^ Die beiden Variablen zur Beziehungsqualität wurden wie folgt dichotomisiert: 0 = sehr schlecht, schlecht oder mittel, 1 = gut oder sehr gut

### Regressionsanalysen.

Zunächst wurden unadjustierte lineare Regressionsanalysen (mit fallweisem Ausschluss bzw. FIML) durchgeführt. Diese Ergebnisse finden sich in Tab. 2. Eine hohe Beziehungsqualität zur Familie ist nicht statistisch signifikant mit depressiven Symptomen assoziiert. Allerdings ist sie statistisch signifikant mit weniger Einsamkeit assoziiert (β = −0,20, *p* < 0,05). Eine hohe Beziehungsqualität zu Freunden ist statistisch signifikant mit weniger depressiven Symptomen (β = −5,89, *p* < 0,001) und weniger Einsamkeit (β = −0,28, *p* < 0,001) assoziiert.

**Tab. 2:** Assoziation von Beziehungsqualität zu Familie und Freunden mit depressiven Symptomen und Einsamkeit bei Personen im Alter von 80 Jahren und älter. Ergebnisse basierend auf Welle 8 der DEAS-Studie (unadjustierte lineare Regressionen)

	Depressive Symptome (Umgang mit fehlenden Werten: fallweiser Ausschluss)	Depressive Symptome (Umgang mit fehlenden Werten: FIML)	Einsamkeit (Umgang mit fehlenden Werten: fallweiser Ausschluss)	Einsamkeit (Umgang mit fehlenden Werten: FIML)
Beziehungsqualität zu Familie: hoch (Referenzkategorie: niedrig)	−1,44 (−5,35 bis 2,48)	−2,52 (−5,88 bis 0,83)	−0,18* (−0,36 bis −0,003)	−0,20* (−0,36 bis −0,05)
Beziehungsqualität zu Freunden: hoch (Referenzkategorie: niedrig)	−5,94** (−9,63 bis −2,26)	−5,89*** (−9,26 bis −2,51)	−0,29*** (−0,46 bis −0,12)	−0,28*** (−0,45 bis −0,12)
R^2^	0,12	0,15	0,07	0,08
Zahl der Beobachtungen	824	861	811	847

Unstandardisierte Beta-Koeffizienten werden präsentiert (95 % Konfidenzintervalle in Klammern), ***p < 0,001, **p < 0,01, *p < 0,05, +*p* < 0,10; Gewichte wurden eingesetzt. FIML: Full Information Maximum Likelihood

Adjustierte Regressionsanalysen (analog aufgebaut) finden sich in Tab. 3 sowie mit vollständiger Darstellung der Kovariaten im Onlinematerial. Eine hohe Beziehungsqualität zur Familie ist hier weder mit depressiven Symptomen noch mit Einsamkeit signifikant assoziiert (die partiellen Eta^2^-Werte betrugen jeweils 0,005). Im Gegensatz dazu ist eine hohe Beziehungsqualität zu Freunden statistisch signifikant mit weniger depressiven Symptomen (β = −3,71, *p* < 0,01) und weniger Einsamkeit (β = −0,28, *p* < 0,01) assoziiert. Die partiellen Eta^2^-Werte lagen hier bei 0,04 (mit depressiven Symptomen als Outcome) bzw. 0,03 (mit Einsamkeit als Outcome).

**Tab. 3:** Assoziation von Beziehungsqualität zu Familie und Freunden mit depressiven Symptomen und Einsamkeit bei Personen im Alter von 80 Jahren und älter. Ergebnisse basierend auf Welle 8 der DEAS-Studie (adjustierte lineare Regressionen)

	Depressive Symptome (Umgang mit fehlenden Werten: fallweiser Ausschluss)	Depressive Symptome (Umgang mit fehlenden Werten: FIML)	Einsamkeit (Umgang mit fehlenden Werten: fallweiser Ausschluss)	Einsamkeit (Umgang mit fehlenden Werten: FIML)
Beziehungsqualität zu Familie: hoch (Referenzkategorie: niedrig)	−1,26 (−3,47 bis 0,95)	−0,96 (−2,76 bis 0,84)	−0,12 (−0,29 bis 0,06)	−0,08 (−0,24 bis 0,08)
Beziehungsqualität zu Freunden: hoch (Referenzkategorie: niedrig)	−3,77** (−6,17 bis −1,38)	−3,71** (−6,02 bis −1,40)	−0,27** (−0,46 bis −0,09)	−0,28** (−0,45 bis −0,10)
R^2^	0,48	0,51	0,22	0,23
Zahl der Beobachtungen	767	810	755	797

Unstandardisierte Beta-Koeffizienten werden präsentiert (95 % Konfidenzintervalle in Klammern), ***p < 0,001, **p < 0,01, *p < 0,05, +*p* < 0,10; Gewichte wurden eingesetzt. Wir haben die folgenden Kovariaten in der Regressionsanalyse berücksichtigt: Alter, Geschlecht, Familienstand, Bildung, Haushaltsnettoäquivalenzeinkommen, Rauchverhalten, Trinkverhalten, Sporthäufigkeit, subjektive Gesundheit und Zahl chronischer Erkrankungen. FIML: Full Information Maximum Likelihood

## Diskussion

Basierend auf Daten der neuesten DEAS-Welle war es Ziel dieser Arbeit, die Assoziation von Beziehungsqualität zu Familie und Freunden mit depressiven Symptomen und Einsamkeit bei Hochaltrigen in Deutschland zu untersuchen. Multiple lineare Regressionen zeigten, dass eine hohe Beziehungsqualität zur Familie weder mit depressiven Symptomen noch mit Einsamkeit signifikant assoziiert ist. Hingegen ist eine hohe Beziehungsqualität zu Freunden statistisch signifikant mit weniger depressiven Symptomen und weniger Einsamkeit assoziiert (mit kleinen bis mittleren Effektgrößen). Diese Studie zu Hochaltrigen erweitert unseren Kenntnisstand in diesem Forschungsfeld, da sich bisherige Studien zu diesen Assoziationen primär auf Kinder und Jugendliche fokussierten [14–16] und vergleichbare Studien im Alter kaum bestehen [17,18]. Frühere Studien bei älteren Populationen haben bspw. gezeigt, dass eine große Distanz zu anderen, seltene Kontaktaufnahme sowie eine geringe Teilnahme am sozialen Leben mit höheren Einsamkeitswerten einhergehen (als Übersicht: [18]). In einigen Studien bei Älteren wurde auch gezeigt, dass eine niedrigere Quantität und Qualität sozialer Beziehungen mit mehr Einsamkeit assoziiert ist (als Übersicht: [18]).

Da familiäre Bindungen häufig lebenslange (und wichtige) Bindungen darstellen, war es für uns zunächst überraschend, dass die Beziehungsqualität zur Familie nicht signifikant mit den Outcomes depressive Symptome und Einsamkeit in den adjustierten Regressionen assoziiert war. Auf den zweiten Blick mögen diese Ergebnisse aber nicht unbedingt überraschen, da die Familie auch zuweilen weniger wichtig sein könnte. Wenn beispielsweise kein gutes Verhältnis zu geografisch weit entfernt lebenden Geschwistern besteht, ist dies vermutlich nicht zwangsläufig belastend, da dies möglicherweise durch andere Faktoren (z. B. qualitativ hochwertige Freundschaften) ausgeglichen werden kann (siehe auch: [33]). Zudem lassen unsere Ergebnisse (fehlende Assoziation) vermuten, dass Personen mit einer schlechten Beziehungsqualität zur Familie den Kontakt möglicherweise nicht vermissen oder sich keinen stärkeren Kontakt wünschen. Familiäre Beziehungen können auch von Verpflichtungen, innerfamiliären Hierarchien und Normen (z. B. bei Geschwisterbeziehungen) geprägt sein [34,35]. Ebenso könnte eine niedrige Beziehungsqualität in diesem Bereich auch schon lange bestehen und die Individuen haben sich inzwischen daran gewöhnt – und es stellt keine Belastung (mehr) für sie dar. Da frühere Forschung (z. B. aus den USA [33]) auf der anderen Seite den Stellenwert der familiären Beziehungen für die sozialen Aktivitäten und die mentale Gesundheit im Alter untermauert, empfehlen wir weitere Forschung (z. B. kulturvergleichend) mit vergleichbaren Tools. Auch künftige Subgruppenanalysen (mit ausreichend großen Subgruppen) wären wünschenswert. Auch ist zu erwähnen, dass sich der Freundeskreis hochaltriger Menschen aufgrund der Mortalität gleichaltriger Freunde immer weiter reduzieren kann, wohingegen in der Familie auch jüngere Mitglieder – wie Kinder und Enkelkinder – vorhanden sind. Künftige Forschung könnte die jeweilige Beziehungsqualität getrennt nach dem Verwandtschaftsverhältnis näher untersuchen. Zudem könnte die jeweilige Entfernung zu den Familienmitgliedern berücksichtigt werden.

Im Gegensatz zur Familie, in die man – von Ausnahmen abgesehen – hineingeboren wird, kann man sich Freundschaften in der Regel aussuchen. Sie zeichnen sich häufig also durch Freiwilligkeit, ähnliche Interessen und auch durch Reziprozität aus [36]. Ebenso ist man in Freundschaften eher selbstbestimmt (im Gegensatz bspw. zu familiären Normen). Insofern ist es gut nachvollziehbar, dass die Beziehungsqualität mit beiden Outcomes signifikant assoziiert war. Qualitativ hochwertige Freundschaften (siehe: [17]) scheinen essenziell für das psychosoziale Wohlbefinden im höchsten Alter zu sein. Vermutlich geben sie den Hochaltrigen gezielt emotionale Unterstützung und die Möglichkeit zur sozialen Integration (siehe auch: [19]). Allerdings müssen diese Zusammenhänge noch vertiefend untersucht werden (z. B. basierend auf qualitativen Verfahren). Hierzu möchten wir künftige Forschungen (z. B. mithilfe von Tiefeninterviews) anregen.

Unsere Ergebnisse sind vor dem Hintergrund einiger Stärken und Einschränkungen zu interpretieren. Welle 8 der bevölkerungsbasierten DEAS-Studie diente als Datengrundlage. Es wurde für wesentliche Kovariaten adjustiert. Auch Modelle mit FIML zur Adressierung fehlender Werte wurden geschätzt. Gewichte wurden eingesetzt. Einschränkend ist insbesondere zu erwähnen, dass die Beziehungsqualität (Familie und Freunde) jeweils anhand von Single-Items operationalisiert wurde. Dies erlaubt eine globale Beurteilung in beiden Bereichen. Allerdings wären künftige Studien wünschenswert, welche die allgemeine Beziehungsqualität vertiefend betrachten (z. B. Konflikthäufigkeit im familiären Setting). Auch die Beziehungsquantität könnte dabei näher untersucht werden. Zudem ist zu berücksichtigen, dass es sich um eine Querschnittstudie handelt, mit den bekannten Limitationen hinsichtlich der Interpretation von Zusammenhängen über die Zeit. So könnten beispielsweise Personen mit stärkerer Einsamkeit bzw. ausgeprägteren depressiven Symptomen im Zeitverlauf auch eine veränderte Beziehungsqualität aufweisen. Darüber hinaus ist davon auszugehen, dass insbesondere vulnerable Gruppen (z. B. mit sehr prekären Familienverhältnissen) im DEAS eher unterrepräsentiert sind.

## Fazit

Unsere Ergebnisse zeigen eine Assoziation einer hohen Beziehungsqualität zu Freunden mit weniger depressiven Symptomen und weniger Einsamkeit bei zu Hause lebenden Hochaltrigen in Deutschland auf. Künftige Studien sollten gezielt die zugrunde liegenden Mechanismen vertiefend beleuchten. Auch wäre es wünschenswert, hochaltrige Personen in institutionalisierten Settings wie Alten- und Pflegeheimen zu betrachten. Ebenso wären Ländervergleiche von Interesse. Zudem wäre es spannend, die Lebensverläufe (über Jahrzehnte) zu betrachten, um vertiefende Einblicke in die Übergänge zu erhalten (z. B. Antizipations- und Habituationseffekte).

## Supplementary Information

Onlinematerial: Adjustierte Regressionsanalysen mit allen Kovariaten

## Data Availability

Die in dieser Studie verwendeten Daten stammen von dem DZA. Die anonymisierten Datensätze der DEAS-Studie stehen für Sekundäranalysen zur Verfügung. Die Daten werden Wissenschaftler:innen an Universitäten und Forschungsinstituten ausschließlich zu wissenschaftlichen Zwecken zur Verfügung gestellt. Weitere Informationen finden sich hier (inkl. Antragsformular): https://www.dza.de/forschung/fdz/datenbestellung/antragsformular (abgerufen am 23.03.2026).
