# Supplementary material for: Quality of relationships among the oldest old in Germany—an examination of the association with depressive symptoms and loneliness
Source: Bundesgesundheitsblatt Gesundheitsforschung Gesundheitsschutz. 2026 Jul 29;69(9):1029–35. [Article in German] doi: 10.1007/s00103-026-04277-9 (PMC13562208; doi:10.1007/s00103-026-04277-9)
Supplement: Supplementary file 1 [file 103_2026_4277_MOESM1_ESM.pdf]

Onlinematerial. Assoziation von Beziehungsqualität zu Familie und Freunden mit depressiven Symptomen und Einsamkeit bei Personen im Alter von 80 Jahren und älter. Ergebnisse basierend auf Welle 8 der DEAS-Studie (adjustierte lineare Regressionen; alle Kovariaten dargestellt).

Variablen	Depressive Symptome (Umgang mit fehlenden Werten: fallweiser Ausschluss)	Depressive Symptome (Umgang mit fehlenden Werten: FIML)	Einsamkeit (Umgang mit fehlenden Werten: fallweiser Ausschluss)	Einsamkeit (Umgang mit fehlenden Werten: FIML)
Beziehungsqualität zu Familie: hoch (Referenzkategorie: niedrig)	-1.26	-0.96	-0.12	-0.08
	(-3.47 - 0.95)	(-2.76 - 0.84)	(-0.29 - 0.06)	(-0.24 - 0.08)
Beziehungsqualität zu Freunden: hoch (Referenzkategorie: niedrig)	-3.77**	-3.71**	-0.27**	-0.28**
	(-6.17 - -1.38)	(-6.02 - -1.40)	(-0.46 - -0.09)	(-0.45 - -0.10)
Alter (in Jahren)	0.02	-0.00	0.00	0.00
	(-0.26 - 0.30)	(-0.26 - 0.26)	(-0.02 - 0.03)	(-0.02 - 0.03)
Geschlecht: Frau (Referenzkategorie: Mann)	-0.34	-0.37	-0.21*	-0.18*
	(-2.27 - 1.59)	(-2.12 - 1.39)	(-0.38 - -0.04)	(-0.33 - -0.02)
Familienstand: - Verheiratet, getrennt lebend (Referenzkategorie: Verheiratet, zusammenlebend)	2.12	1.73	0.08	0.08
	(-1.49 - 5.73)	(-1.76 - 5.23)	(-0.24 - 0.40)	(-0.22 - 0.37)
- Geschieden	2.83+	2.90+	0.24*	0.23*
	(-0.49 - 6.15)	(-0.17 - 5.96)	(0.02 - 0.45)	(0.01 - 0.44)
- Verwitwet	3.23**	3.04***	0.19*	0.17*
	(1.17 - 5.29)	(1.27 - 4.80)	(0.00 - 0.38)	(0.00 - 0.33)
- Ledig	0.37	0.09	-0.09	-0.10
	(-3.55 - 4.30)	(-3.74 - 3.93)	(-0.57 - 0.38)	(-0.54 - 0.35)
Bildungsniveau nach ISCED-97: - Mittel (Referenzkategorie: Niedrig)	0.99	1.10	0.10	0.09
	(-1.04 - 3.02)	(-0.79 - 2.99)	(-0.09 - 0.29)	(-0.09 - 0.27)
- Hoch	2.02+	2.27*	0.03	0.05
	(-0.00 - 4.04)	(0.37 - 4.17)	(-0.19 - 0.25)	(-0.16 - 0.27)
Haushaltsnettoeinkommen in Euro	-0.00	-0.00+	-0.00	-0.00
	(-0.00 - 0.00)	(-0.00 - 0.00)	(-0.00 - 0.00)	(-0.00 - 0.00)
Derzeitiges Rauchverhalten: Ja, gelegentlich (Referenzkategorie: Ja, täglich)	-0.61	-1.78	0.57+	0.63*
	(-5.03 - 3.82)	(-6.01 - 2.45)	(-0.11 - 1.24)	(0.03 - 1.23)
- Nein, nicht mehr	2.06	0.96	0.19	0.24
	(-1.15 - 5.27)	(-2.13 - 4.06)	(-0.33 - 0.71)	(-0.19 - 0.68)
- Habe noch nie geraucht	3.58*	2.44	0.27	0.32
	(0.58 - 6.58)	(-0.49 - 5.37)	(-0.26 - 0.80)	(-0.13 - 0.76)
Sport machen: Mehrmals pro Woche (Referenzkategorie: Täglich)	-0.61	-0.45	-0.07	-0.09
	(-2.42 - 1.20)	(-2.16 - 1.25)	(-0.35 - 0.20)	(-0.36 - 0.18)
- Einmal pro Woche	-0.23	-0.82	-0.02	0.00
	(-2.75 - 2.30)	(-3.17 - 1.52)	(-0.39 - 0.35)	(-0.34 - 0.34)
- 1 bis 3mal im Monat	-2.81+	-2.67+	-0.24	-0.24+
	(-5.70 - 0.08)	(-5.43 - 0.09)	(-0.52 - 0.05)	(-0.51 - 0.04)
- Seltener	0.81	0.71	-0.20	-0.23+
	(-1.89 - 3.51)	(-1.71 - 3.13)	(-0.45 - 0.06)	(-0.49 - 0.04)
- Nie	0.20	0.17	-0.06	-0.06
	(-1.65 - 2.05)	(-1.61 - 1.95)	(-0.32 - 0.21)	(-0.31 - 0.20)
Häufigkeit von alkoholischen Getränken: Mehrmals pro Woche (Referenzkategorie: Täglich)	1.73	1.75	0.20	0.15
	(-0.87 - 4.32)	(-0.63 - 4.13)	(-0.16 - 0.57)	(-0.19 - 0.50)
- Einmal pro Woche	1.14	1.16	0.06	0.04
	(-1.48 - 3.75)	(-1.32 - 3.64)	(-0.25 - 0.38)	(-0.26 - 0.34)
- 1 bis 3mal im Monat	-0.24	-0.20	0.01	0.01
	(-2.71 - 2.23)	(-2.47 - 2.07)	(-0.30 - 0.31)	(-0.27 - 0.30)
- Seltener	0.46	0.69	-0.01	-0.04
	(-1.98 - 2.90)	(-1.65 - 3.03)	(-0.32 - 0.30)	(-0.33 - 0.26)
- Nie	1.37	1.08	0.16	0.14
	(-1.72 - 4.47)	(-1.73 - 3.89)	(-0.15 - 0.47)	(-0.16 - 0.43)
Subjektiver Gesundheitszustand (von 1 = sehr gut bis 5 = sehr schlecht)	4.08***	4.14***	0.05	0.05
	(3.02 - 5.15)	(3.16 - 5.12)	(-0.05 - 0.15)	(-0.05 - 0.14)
Anzahl körperlicher Erkrankungen	-0.16	-0.15	-0.02	-0.02
	(-0.55 - 0.24)	(-0.52 - 0.22)	(-0.05 - 0.01)	(-0.05 - 0.01)
Konstante	-4.91	-1.82	1.60	1.75
	(-28.92 - 19.09)	(-23.64 - 20.01)	(-0.50 - 3.70)	(-0.34 - 3.83)
Zahl der Beobachtungen	767	810	755	797
R^2	0.48	0.51	0.22	0.23

Unstandardisierte Beta-Koeffizienten werden präsentiert (95% Konfidenzintervalle in Klammern), *** p<0.001, ** p<0.01, * p<0.05, + p<0.10; Gewichte wurden eingesetzt.
